# *Neisseria gonorrhoeae O*-linked pilin glycosylation: functional analyses define both the biosynthetic pathway and glycan structure

**DOI:** 10.1111/j.1365-2958.2007.05806.x

**Published:** 2007-08-01

**Authors:** Finn Erik Aas, Åshild Vik, John Vedde, Michael Koomey, Wolfgang Egge-Jacobsen

**Affiliations:** 1 Centre for Molecular Biology and Neuroscience 0316 Oslo, Norway.; 2 Department of Molecular Biosciences 0316 Oslo, Norway.; 3 Department of Chemistry, University of Oslo 0316 Oslo, Norway.

## Abstract

*Neisseria gonorrhoeae* expresses an *O*-linked protein glycosylation pathway that targets PilE, the major pilin subunit protein of the Type IV pilus colonization factor. Efforts to define glycan structure and thus the functions of pilin glycosylation (Pgl) components at the molecular level have been hindered by the lack of sensitive methodologies. Here, we utilized a ‘top-down’ mass spectrometric approach to characterize glycan status using intact pilin protein from isogenic mutants. These structural data enabled us to directly infer the function of six components required for pilin glycosylation and to define the glycan repertoire of strain N400. Additionally, we found that the *N. gonorrhoeae* pilin glycan is *O*-acetylated, and identified an enzyme essential for this unique modification. We also identified the *N. gonorrhoeae* pilin oligosaccharyltransferase using bioinformatics and confirmed its role in pilin glycosylation by directed mutagenesis. Finally, we examined the effects of expressing the PglA glycosyltransferase from the *Campylobacter jejuni N*-linked glycosylation system that adds *N*-acetylgalactosamine onto undecaprenylpyrophosphate-linked bacillosamine. The results indicate that the *C. jejuni* and *N. gonorrhoeae* pathways can interact in the synthesis of *O*-linked di- and trisaccharides, and therefore provide the first experimental evidence that biosynthesis of the *N. gonorrhoeae* pilin glycan involves a lipid-linked oligosaccharide precursor. Together, these findings underpin more detailed studies of pilin glycosylation biology in both *N. gonorrhoeae* and *N. meningitidis*, and demonstrate how components of bacterial *O*- and *N*-linked pathways can be combined in novel glycoengineering strategies.

## Introduction

Protein glycosylation plays important roles in biological systems not only because it is one of the major post-translational modifications, but also because it has significant effects on protein properties and functions. Recently, heightened attention has been drawn towards protein glycosylation in bacteria primarily because of the increasing frequency with which it is seen in pathogenic species (Benz and Schmidt, 2002; Szymanski and Wren, 2005). In particular, most glycoproteins of bacterial pathogens are either surface localized or trafficked for secretion and appear to influence interactions with the host. Prime examples among Gram-negative species include flagellins present in *Pseudomonas aeruginosa* (Brimer and Montie, 1998), *Helicobacter pylori* (Schirm *et al*., 2003) and *Campylobacter jejuni* flagella (Doig *et al*., 1996), pilin subunits of *P. aeruginosa* (Castric *et al*., 2001) and neisserial type IV pili (Tfp) (Stimson *et al*., 1995), the *Escherichia coli* TibA (Lindenthal and Elsinghorst, 2001) and AIDA-I autotransporter adhesins (Benz and Schmidt, 2001), and the *Haemophilus influenzae* HMW1 adhesin (Grass *et al*., 2003). In many instances, glycosylation-defective mutants have been shown to be attenuated in virulence-associated properties and colonization (Szymanski *et al*., 2002; Grass *et al*., 2003; Schirm *et al*., 2003; Hendrixson and DiRita, 2004; Arora *et al*., 2005). Nonetheless, the full significance of protein glycosylation has yet to be precisely defined in any prokaryotic system.

Tfp are a unique class of filamentous appendages defined by their conserved biogenesis pathway, structure and associated properties. They play important roles in prokaryotic cell biology by influencing social behaviour and by being required for motility, phage susceptibility, conjugation and DNA uptake during natural genetic transformation in many instances. Tfp also contribute to colonization of mucosal surfaces by many human pathogens, including *Neisseria gonorrhoeae*, *N. meningitidis*, *Vibrio cholerae*, *P. aeruginosa* and enteropathogenic strains of *E. coli* (Craig *et al*., 2004). Neisserial Tfp function in this capacity by promoting microbial adherence to epithelial cells and the ability of bacterial cells to aggregate into multicellular infectious units. Many observations suggest that both properties can be influenced by the structure of the pilin protein subunits as well as ancillary protein factors (Jonsson *et al*., 1994; Wolfgang *et al*., 1998; Rytkonen *et al*., 2001; Winther-Larsen *et al*., 2001; 2005). In-depth knowledge of Tfp pilin structure and chemistry is therefore crucial to unravelling the molecular mechanisms underlying these processes. In the cases of *N. gonorrhoeae* and *N. meningitidis* Tfp, this situation is made more complicated by the extensive changes in PilE primary structure accompanying antigenic variation (Virji and Heckels, 1984; Zhang *et al*., 1992).

Covalent post-translational modifications of Tfp pilin subunits also provide sources for structural complexity and diversification. PilE, the protein subunit of *N. gonorrhoeae* and *N. meningitidis* Tfp, is also glycosylated with *N. gonorrhoeae* strain N400 subunits bearing a disaccharide composed of a hexose residue linked to a proximal 2,4-diacetamido-2,4,6-trideoxyhexose sugar (HexDATDH) at serine 63 (S^63^) (Hegge *et al*., 2004) and *N. meningitidis* strain C311 pilin bearing the trisaccharide Gal(1–4) Gal(1–3) 2,4-diacetamido-2,4,6-trideoxyhexose (DATDH) at a serine or threonine between residues 50 and 73 (Stimson *et al*., 1995). As shown by mass spectrometric (MS) analyses of PilE from defined pilin glycosylation (*pgl*) mutants, modification of *N. gonorrhoeae* PilE with the HexDATDH glycan requires the products of at least four genes (Hegge *et al*., 2004). The products of the *N. gonorrhoeae pgIC* and *pglD* genes are structurally related to sugar transaminases and dehydratases respectively, and are similar to those implicated in the biosynthesis of the proximal bacillosamine component of *N*-linked glycans in *C. jejuni* glycoproteins (Weerapana and Imperiali, 2006). The masses for *N. gonorrhoeae* and *N. meningitidis* pilin DATDH are consistent with bacillosamine although sugar stereochemistries have not been resolved. Like mutations in *pgIC* and *pglD*, mutations in *pglF* result in the recovery of non-glycosylated peptides derived from PilE (Hegge *et al*., 2004). PglF has been inferred to be specifically involved in membrane translocation or flipping of a lipid-attached carbohydrate (Kahler *et al*., 2001; Power *et al*., 2000). However, there is no direct evidence that the neisserial *pgl* pathways involve such a precursor intermediate. Addition of sugar to the basal DATDH sugar appears to require PglA (also termed PgtA), as MS studies of pilin from *pglA*-null mutants revealed the presence of only the DATDH sugar in one study (Hegge *et al*., 2004) and a mass loss of 162 Da in another (Banerjee *et al*., 2002). Furthermore, overexpression of PglA in *E. coli* led to an activity incorporating radiolabelled UDP-Gal into lipid-linked fractions (Banerjee *et al*., 2002). Corresponding *N. meningitidis pgl* genes have been characterized with mutations at these loci altering PilE biochemical properties compatible with altered carbohydrate composition (migration in SDS-PAGE, chemical reactivity, recognition by lectins and carbohydrate-specific antibodies, sugar GC-MS composition profiles of purified pili, etc.) (Jennings *et al*., 1998; Power *et al*., 2000; 2003; Kahler *et al*., 2001). The potential significance of PilE glycan modifications remains unclear, as dramatic phenotypic alterations have not been seen in either *pgl* mutants or pilin missense mutants incapable of being glycosylated (Marceau *et al*., 1998; Marceau and Nassif, 1999). In *P. aeruginosa* strain 1244, an *O*-linked glycan moiety composed of a single unit of endogenous O-antigen is attached to the PilA pilin C-terminal serine residue (DiGiandomenico *et al*., 2002). A mutant expressing unmodified PilA (due to the absence of PilO oligosaccharyltransferase) was reported to be phenotypically similar to its wild-type progenitor, although subtle defects in twitching motility and mouse colonization were seen (Smedley *et al*., 2005).

Because MS measures the molecular weight, it is the method of choice for the detection and characterization of post-translational modifications, and potentially can identify any covalent modification that alters the mass of a protein. The MS data for most PilE covalent modifications have been generated by a ‘bottom-up’ approach in which proteolytically derived peptides were examined by liquid chromatography electrospray ionization (ESI) tandem MS [collision-induced dissociation (CID) MS/MS] analysis. While this peptide-based MS is a sensitive and powerful tool for identifying post-translational modifications, it also has its limitations. For example, several peptides, and especially those with post-translational modifications of a given protein digest, may escape detection due to intrinsic properties relating to MS ionization efficiency, extreme hydrophilicity/hydrophobicity or low stoichiometric abundance, and certain post-translational modifications may exhibit selective instability during sample preparation. As such, complete characterization of the PilE glycan may have yet to be realized.

To explore the *N. gonorrhoeae* pilin glycan in more detail, pilus/pilin purification schemes and ESI-MS methodologies were developed so that intact PilE protein species could be analysed. In addition to confirming and extending our earlier findings derived from a ‘bottom-up’ approach, we present here important new information with respect to PilE glycosylation. Specifically, we identify the pilin oligosaccharyltransferase and demonstrate that the pilin glycan repertoire of strain N400 is structurally more complex than previously realized.

## Results

### Mass spectrometric analysis of intact PilE from defined *pgl* mutant backgrounds

**Fig. 1:**
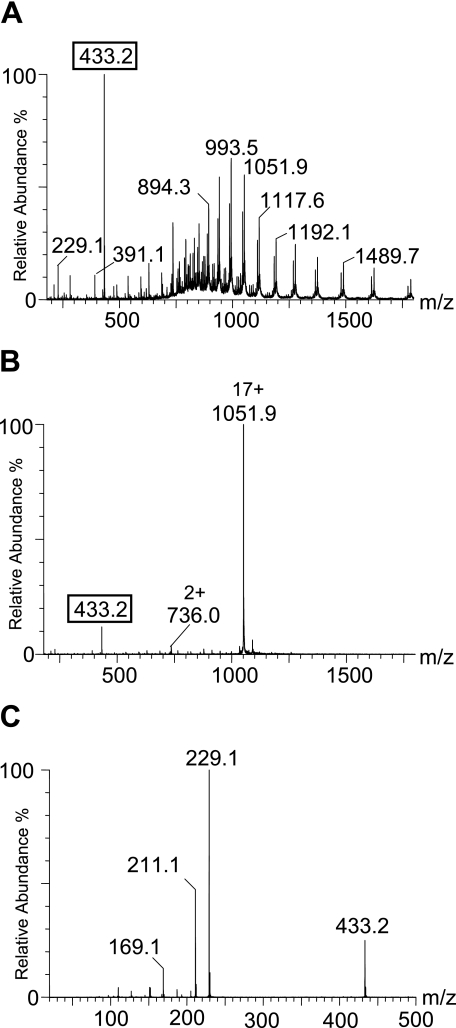
Electrospray infusion MS analyses of intact PilE from *N. gonorrhoeae* strain N400 (wt, wild-type) A. ESI mass spectrum over a range of 180–1800 *m/z*. The major glycan oxonium ion species (*m/z* 433.2) is boxed. Additional oxonium ions for HexDATDH (*m/z* 391.1) and DATDH (*m/z* 229.1) are also detectable. B. CID MS/MS analysis of the multiple charged PilE species [M + 17H]^17+^ at *m/z* 1051.9 seen in panel A shows mainly cleavage of the glycosidic bond between glycan and polypeptide, yielding predominantly the signal at *m/z* 433.2 (boxed) for the corresponding oxonium ion. C. CID MS/MS analysis of the major oxonium ion species at *m/z* 433.2 generated by in-source fragmentation.

In order to better characterize PilE glycan modification in *N. gonorrhoeae*, we examined intact pilin derived from *pgl* backgrounds using a ‘top-down’ MS approach recently detailed by us and others (Schirm *et al*., 2005; Aas *et al*., 2006). Not only did this technique allow PilE molecular weight determination, it also enabled detection of characteristic oxonium ions arising from the cleavage of glycosidic bonds during in-source fragmentation. As shown previously, the conventional ESI mass spectrum of PilE in purified Tfp from the wild-type strain shows a broad envelope of multiply charged ions, extending over a range of 500–1700 *m/z*, as well as the presence of a prominent signal at *m*/*z* 433.2 and those corresponding to known oxonium ions for DATDH and HexDATDH at *m*/*z* 229.1 and 391.2 respectively (Fig. 1A). As these species were not seen in MS analysis of PilE carrying an alanine substitution for serine at residue 63 (the site of glycan occupancy) (Aas *et al*., 2006), all three are associated with PilE glycosylation. In addition, covalent modification of PilE with this glycan moiety is supported by the readily detectable oxonium ion at *m/z* 433.2 in tandem MS analysis (CID MS/MS) of the multiply charged precursor ions of PilE at *m/z* 1051.9 [M + 17H]^17+^ (Fig. 1B). The second-generation fragment ion spectrum (CID MS/MS analysis of in-source fragmentation generated ions) of the oxonium ion at *m/z* 433.2 revealed its fragmentation into DATDH (*m*/*z* 229.1), together with further DATDH fragment signals at *m/z* 211.1 (loss of water) and at *m/z* 169.1 (loss of water plus loss of one *N*-linked acetyl group) respectively (Fig. 1C). Accordingly, the species at *m*/*z* 433.2 is an oxonium ion of HexDATDH bearing an additional mass substituent of 42 Da.

**Fig. 2:**
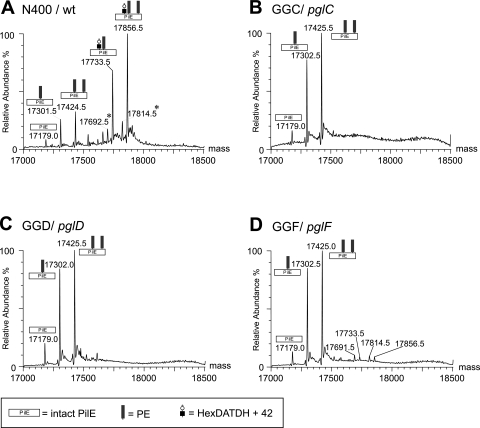
The products of *pgIC*, *pglD* and *pglF* are required for pilin glycosylation. Deconvoluted molecular weight spectra from intact PilE ESI-MS analyses A. Strain N400 (wt, wild-type). B. Strain GGC (*pgIC*::*kan*). C. Strain GGD (*pglD*::*kan*). D. strain GGF (*pglF*::*kan*). The two predominant peaks in (A), at *m/z* 17733.5 and *m/z* 17856.5, correlate with PilE species carrying the 432.2 Da glycan (HexDATDH plus 42 Da) in conjunction with one or two PE moieties, while the two peaks marked with an asterisk in (A) at *m/z* 17692.5 and *m/z* 17814.5 correlate with PilE species being modified with HexDATDH in conjunction with one or two PE groups. The two predominant peaks in (B, C, D) correlate with PilE species only carrying one or two PE groups. Note that small amounts of glycosylated pilin are detectable in the *pglF* mutant; see Fig. S1 containing conventional MS spectra of corresponding strains. A complete list of all oxonium ion species with *m/z*-values and corresponding molecular weight values of all PilE species are found in Table S1.

The reconstructed molecular mass profile of the corresponding wild-type PilE spectrum revealed a broad distribution of modified forms, with the most prevalent species observed corresponding to the protein modified with a single 432.2 Da glycan moiety (HexDATDH modified with an unknown moiety of 42 Da) and either one or two phosphoethanolamine (PE) moieties at *m*/*z* 17733.5 and *m*/*z* 17856.5 (Fig. 2A). Additional modified PilE forms were also observed that could be assigned to PilE differentially modified with one or two PE moieties alone and in combination with a DATDH or the HexDATDH glycan moiety. We then examined PilE from *pgIC-, pglD-* and *pglF-*null backgrounds. The products of the *pgIC* and *pglD* genes are related to sugar aminotransferases and dehydratases implicated in the biosynthesis of the DATDH sugar, whereas that of *pglF* is related to proteins implicated in the membrane translocation of a lipid-attached carbohydrate (Power *et al*., 2000). Here, conventional ESI mass spectra from *pgIC* and *pglD* mutants were devoid of oxonium ions (Fig. S1). Moreover, the reconstructed molecular mass spectra of each indicated a limited distribution of PilE modified forms at *m*/*z* 17302 and *m*/*z* 17425, accounted for solely by the presence of one or two PE modifications with a minor signal at *m*/*z* 17179 reflecting unmodified polypeptide (Fig. 2B and C). PilE derived from the *pglF*-null background resembled that from the *pgIC* and *pglD* backgrounds with the exception that low-level signals corresponding to glycosylated forms were seen in the molecular weight spectrum (Fig. 2D). Likewise, oxonium ions signals at *m/z* 433.2 were detectable in conventional ESI mass spectrum, albeit at reduced levels (Fig. S1D). These findings are most consistent with the hypothesis that there is a PglF-independent mechanism for glycan delivery to the oligosaccharyltransferase reaction that functions inefficiently. As the *pglF* allele employed a simple gene cassette insertion at residue 119 of the open reading frame (ORF), we cannot, however, formally rule out the possibility that the ensuing polypeptide retains partial activity.

**Fig. 3:**
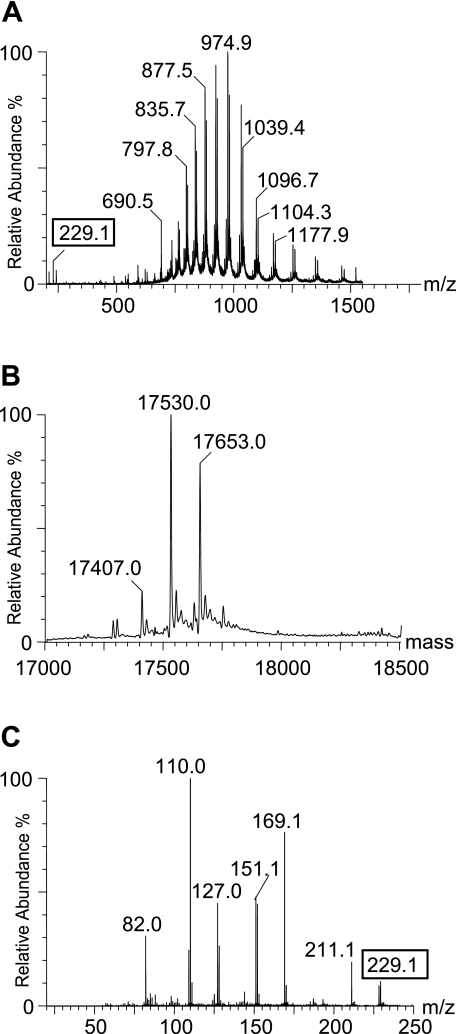
The product of *pglA* is required for disaccharide modification of PilE A. ESI mass spectrum over a range of 180–1800 *m/z*; strain KS141 (*pglA*::*kan*). The major oxonium ion species detected is boxed; DATDH (*m/z* 229.1). B. Deconvoluted molecular weight spectrum from intact PilE ESI-MS analyses. The two major peaks at *m/z* 17530.0 and *m/z* 17653.0 represent PilE modified with DATDH in conjunction with one and two PE groups respectively. C. CID MS/MS spectrum of the major oxonium ion species at 229.1 *m/z* (boxed) generated by in-source fragmentation. A complete list of all oxonium ion species with *m/z*-values and corresponding molecular weight values of all PilE species are found in Table S1.

The conventional ESI mass spectrum of PilE derived from a *pglA*-null mutant revealed the presence of only the DATDH oxonium ion at *m/z* 229.1, while its deconvoluted molecular mass spectrum showed a restricted distribution of PilE forms accounted for solely by modifications with the DATDH sugar in conjunction with the PE substituents (Fig. 3A and B). Moreover, the second-generation fragment ion spectrum of the DATDH oxonium ion at *m/z* 229.1 showed its characteristic side-chain fragmentation, including the loss of water/ammonia (Fig. 3C), as described earlier (Soo *et al*., 2004). Also, there was no evidence here for the anomalous substituent with a mass of 42 Da. Consistent with previous data (Hegge *et al*., 2004), the role of PglA is therefore most simply ascribed to its activity as a glycosyltransferase that adds the hexose onto the basal DATDH sugar.

### PglE is required for the expression of the trisaccharide form of the PilE glycan

**Fig. 4:**
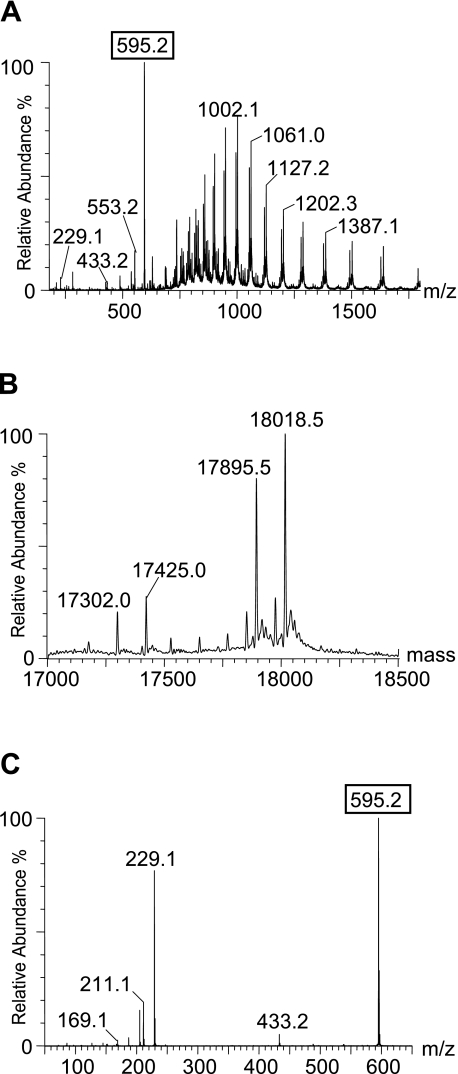
The product of *pglE* is required for trisaccharide modification of PilE A. ESI mass spectrum over a range of 180–1800 *m/z*; strain KS142 (*pglE*_*on*_). The major oxonium ion species detected is boxed; DATDH (*m/z* 595.2). B. Deconvoluted molecular weight spectrum from intact PilE ESI-MS analyses. The two major peaks at *m/z* 17895.5 and *m/z* 18018.5 represent PilE modified with the 594.2 Da glycan in conjunction with one and two PE groups respectively. C. CID MS/MS spectrum of the major oxonium ion species at 595.2 *m/z* (boxed) generated by in-source fragmentation. A complete list of all oxonium ion species with *m/z*-values and corresponding molecular weight values of all PilE species are found in Table S1.

Based on indirect data, previous work in *N. meningitidis* has suggested that the *pglE* gene product is involved in the expression of a trisaccharide glycan form (Power *et al*., 2003). PglE has been proposed to undergo phase-variable expression in both *N. gonorrhoeae* and *N. meningitidis* due to the contraction/expansion of the repetitive sequence element 5′-CAAACAA-3′ (and its variants) early in its ORF. In the strain of *N. gonorrhoeae* used here, the *pglE* ORF is in its off configuration (having 15 copies of the 5′-CAAACAA-3′ repeat) (Power *et al*., 2003). To investigate the potential influence of PglE on PilE glycosylation in *N. gonorrhoeae*, we engineered the *pglE* allele in this strain to be in its ‘on’ configuration. As shown in Fig. 4A, two new species at *m*/*z* 595.2 and *m*/*z* 553.2 were detected in the lower end of the conventional ESI mass spectrum of PilE from this background. These signals were most consistent with oxonium ions derived from a HexHexDATDH glycan with and without the anomalous 42 Da substituent. The second-generation fragment ion spectrum of the species at *m*/*z* 595.2 showed its fragmentation into two oxonium fragment ions at *m*/*z* 433.2 and *m*/*z* 229.1 respectively, indicating its composition of one DATDH and two hexose molecules (Fig. 4C). Moreover, these data indicated that the anomalous 42 Da moiety was associated with the hexose proximal to the DATDH sugar. This is the first direct evidence based on glycan structure that PglE acts as glycosyltransferase necessary for the addition of the second hexose to make the HexHexDATDH trisaccharide. Furthermore, this *pglE*_*on*_ allele had no effect on glycan structure in a *pglA* background, showing that *pglE* maps genetically downstream of *pglA* (Fig. S2).

### Identification and characterization of the PglO pilin oligosaccharyltransferase

**Fig. 5:**
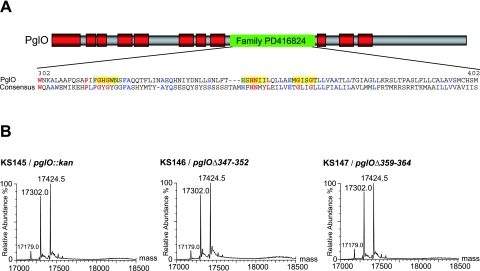
PglO in-frame deletions encompassing residues conserved in Prodom domain PD416824 abolish pilin glycosylation A. Upper part. Graphical overview of the domain structure of PglO. Colour codes: red, transmembrane regions (residues 1–35, 45–62, 69–87, 102–124, 131–153, 202–221, 226–241, 251–273, 366–377, 405–424 and 436–458); green, ProDom family PD416824 (residues 302–402). Lower part. Alignment of the PD416824 domain of PglO with the consensus sequence of the ProDom family. Colour codes: red, residues highly conserved throughout the whole ProDom family PD416824; blue, residues conserved between PglO and the PD416824 consensus sequence; yellow, residues deleted in the *pglO* in-frame deletion mutants. B. Deconvoluted molecular weight spectra from intact PilE ESI-MS analyses. Left panel. strain KS145 (*pglO*::*kan*); Middle panel. strain KS146 (*pglO*_Δ347−352_); Right panel. KS147 (*pglO*_Δ359−364_). A complete list of all oxonium ion species with *m/z*-values and corresponding molecular weight values of all PilE species are found in Table S1.

In efforts to identify the enzyme responsible for glycan transfer to gonococcal PilE, we noted that Tfp pilin subunits in some strains of *P. aeruginosa* are modified by an oligosaccharide structurally identical to the O-antigen repeat unit (Castric *et al*., 2001). Based on its requirement for pilin O-antigen modification, the *P. aeruginosa* PilO protein has been modelled as an oligosaccharyltransferase mediating the commited step of the pathway (Castric, 1995). This idea is further supported by the structural similarities of PilO to the O-antigen ligase family as defined by the presence of the Prodom PD416824 domain as well as the Pfam domain PF04932, together with the presence of multiple, membrane-spanning domains. The molecular mechanism by which this family of proteins delivers oligosaccharide from undecaprenyl-linked intermediates to substrates has not been resolved. Attempts to find ORFs in the neisserial genome sequences with sequence identities to PilO using a variety of algorithms were unsuccessful. However, the FA1090 genome sequence carries an ORF designated NG0178 possessing the PD416824 and PF04932 domains despite the fact that this species does not express O-antigen (Fig. 5A). Moreover, this ORF shared strong similarities to *P. aeruginosa* PilO in its organization of putative membrane-spanning domains (data not shown). To test the potential role of this ORF in pilin glycosylation, we constructed four defined mutations, including one kan^R^ gene cassette insertion and three, short in-frame deletions designed to disrupt regions of the PD416824 domain bearing highly conserved residues. PilE was then purified from these mutants and examined by MS. As shown in Fig. 5B, the results for each were identical to those seen for the *pgIC-* and *pglD*-derived samples showing the absence of signals corresponding to a PilE-associated glycan.

### Pilin glycan *O*-acetylation and the role of PglI

**Fig. 6:**
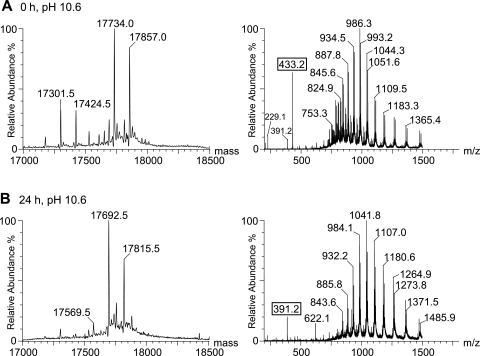
A 42 Da substituent is lost from the *m/z* 433.2 pilin glycan over time during incubation at high pH. PilE from strain N400 (wild-type) was incubated at pH 10.6 and analysed immediately and after 24 h of incubation A. At 0 h incubation, the two major peaks in the deconvoluted molecular weight spectrum from intact PilE ESI-MS analyses (left panel), correlate with PilE species being modified with the 432.2 Da glycan in conjunction with one or two PE groups. Right panel shows the oxonium ions for DATDH (*m/z* 229.1), HexDATDH (*m/z* 391.2) and the *m/z* 433.2 species (boxed, proposed acHexDATDH). B. After 24 h of incubation, the two major peaks in the deconvoluted molecular weight spectrum (left panel) correlate with PilE species being modified with HexDATDH in conjunction with one or two PE groups. *Right panel* shows the oxonium ion for HexDATDH (*m/z* 391.2; boxed). A complete list of all oxonium ion species with *m/z*-values and corresponding molecular weight values of all PilE species are found in Table S1.

**Fig. 7:**
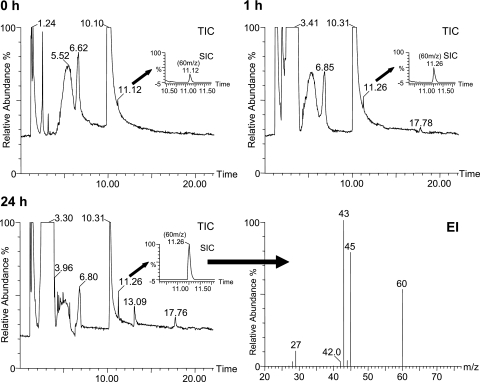
Detection of acetic acid in the supernatants of an alkaline incubation mixture of Tfp preparations. Total ion chromatograms (TIC) of the supernatant of the alkaline incubation mixtures of PilE at pH 10.5 after 0, 1 and 24 h (insets with selected ion chromatograms (SIC) of *m/z* 60 for acetic acid). Electron impact ionization (EI) spectrum of the compound eluting at 11.2 min confirms formation of acetic acid over time.

Mass spectrometric analysis of intact wild-type PilE demonstrated an additional glycan modification with a mass of 42 Da associated with the presence of the proximal hexose that could be most readily ascribed to acetylation, a modification not uncommon in bacterial lipopolysaccharide (LPS) and capsular polysaccharides (CPS). In fact, *O*-acetyl modifications of peptidoglycan, LPS and CPS have been well characterized in *N. meningitidis* (Antignac *et al*., 2003; Gudlavalleti *et al*., 2004; Kahler *et al*., 2006). *O*-acetyl groups on polysaccharides can be selectively hydrolysed and released as acetate under mild alkaline conditions. We therefore examined the effects of this treatment on intact PilE derived from the wild-type background. As shown in Fig. 6, prolonged incubation of wild-type pili in high-pH buffer led to a shift in the predominant oxonium ion seen in conventional MS from *m/z* 433.2 to *m/z* 391.2, and the reconstructed molecular mass spectra revealed a corresponding shift of the predominant species by a mass unit of 42 Da. The release of acetic acid from PilE preparations under alkaline treatment after 0, 1 and 24 h was confirmed by performing gas-chromatography-MS (GC-MS) analyses with electron impact ionization from supernatants. These GC-MS analyses unambiguously confirmed the formation of acetic acid by comparing retention times and electron impact spectra with that of the authentic standard of acetic acid (Fig. 7, data not shown). Additional semiquantitative analyses based on comparisons of the relative peak intensities of molecular ion signals of acetic acid at 60 *m/z* and 11.2 min retention time [insets of Fig. 7, selected ion chromatograms (SIC)] detected in total ion chromatograms confirmed increased release of acetic acid over time.

**Fig. 8:**
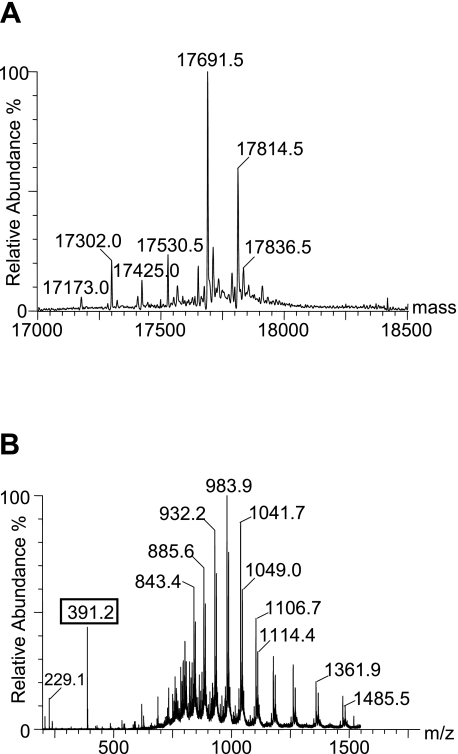
The product of *pglI* is required for acetylation of the PilE glycan A. Deconvoluted molecular weight spectrum from intact PilE ESI-MS analysis showing the pilin species found in a *pglI*-null mutant (strain KS144, *pglI*::*kan*). The two major peaks at 17 691.5 and 17 814.5 Da represent PilE modified with HexDATDH in conjunction with one and two PE groups respectively. B. ESI mass spectrum over a range of 180–1800 *m/z*. Oxonium ions for DATDH (*m/z* 229.1) and HexDATDH (*m/z* 391.2; boxed) are seen. A complete list of all oxonium ion species with *m/z*-values and corresponding molecular weight values of all PilE species are found in Table S1.

To further substantiate *O*-acetylation of the pilin glycan, we scanned the FA1090 genome sequence for ORFs sharing identity to known glycan *O*-acetylases, and identified NG0065 and NG1710 as likely candidates. NGO0065 is orthologous to *N. meningitidis* PglI proposed to be involved in the biosynthesis of the basal sugar residue of the pilin-linked glycan in that species (Warren *et al*., 2004), while NG1710 is orthologous to Lot3 that is required for *O*-acetylation of the terminal *N*-acetylglucosamine of the *Neisseria meningitidis* LPS inner core (Kahler *et al*., 2006). We constructed a NG0065/*pglI*-null allele in strain N400 and found that in the conventional ESI mass spectrum of intact PilE from this background, oxonium ion signals corresponded to species derived solely from a HexDATDH disaccharide (Fig. 8). Moreover, the reconstructed molecular mass profile was dominated by signals corresponding to PilE modified with the HexDATDH glycan in its single- and double-PE modified forms. To confirm that *pglA* is epistatic to *pglI*, intact protein analysis was carried out on PilE from a *pglA*, *pglI* background, and the results were identical to those seen for the *pglA* mutant sample (Fig. S3). The MS data thus demonstrate that the proximal hexose of the PilE glycan is *O*-acetylated, while the genetic data implicate the product of *pglI* in this modification. In the absence of complementation, however, we cannot conclusively determine whether it is the lack of functional PglI, or the altered expression of other genes, that accounts for the phenotype. Whatever the case, this is, to our knowledge, the first time that protein glycan *O*-acetylation has been documented in prokaryotes.

### Complementation of a *N. gonorrhoeae pglA* mutant by *C. jejuni* PglA

**Fig. 9:**
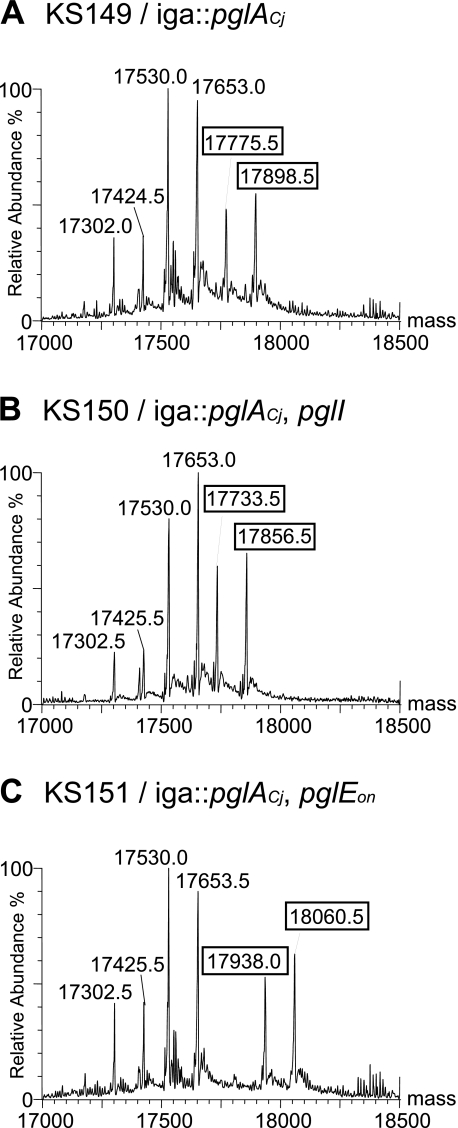
*C. jejuni pglA* complements a *pglA*-null mutant in *N. gonorrhoeae.* Deconvoluted molecular weight spectra from intact PilE ESI-MS analyses. Peaks representing novel PilE species are boxed in each panel A. Strain KS149 (*pglA*::*kan*, *iga*::*pglA*_*Cj*_). B. Strain KS150 (*pglA*::*kan*, *iga*::*pglA*_*Cj*_, *pglI*_*fs*_). C. Strain KS151 (*pglA*::*kan*, *iga*::*pglA*_*Cj*_*, pglE*_*on*_). A complete list of molecular weight values of all PilE species are found in Table S1.

The early steps in the neisserial pilin glycosylation pathway appear to be very similar to those responsible for *N*-linked general protein glycosylation in *C. jejuni* (Weerapana and Imperiali, 2006). In the neisserial *O*-linked systems, however, it remains unknown whether glycan synthesis and extension takes place on membrane-associated lipid carrier (with an oligosaccharide subsequently being delivered *en bloc*), or whether it occurs by step-wise addition of monosaccharides onto pilin (as typified by eukaryotic systems). To address this matter, the effects of expressing *C. jejuni* PglA that transfers *N*-acetylgalactosamine (GalNAc) from uridine diphosphate (UDP)-linked precursor to undecaprenylpyrophosphate-linked bacillosamine (Glover *et al*., 2005) were examined in a *N. gonorrhoeae pglA* background. *C. jejuni pglA* complemented the *N. gonorrhoeae* mutant, as seen by expression of novel pilin form whose conventional and deconvoluted MS profiles were most consistent with the presence of an acetylated HexNAcDATDH glycan, albeit not in stoichiometric amounts (Fig. 9A and Fig. S4A – oxonium ion signal at *m/z* 474.2). Acetylation of this unique glycan form was confirmed by the mass 42 Da reduction seen in this background in combination with the *pglI*-null allele (Fig. 9B and Fig. S4B – oxonium ion signal at *m/z* 432.2). Furthermore, *pglA*_*Cj*_ complementation in a *N. gonorrhoeae pglA*, *pglE*_*on*_ background resulted in the expression of pilin with an *O*-acetylated, HexHexNAcDATDH trisaccharide (Fig. 9C and Fig. S4C – oxonium ion signal at *m/z* 636.2). These findings strongly suggest that the neisseria pathway is capable of utilizing an undecaprenylpyrophosphate-linked disaccharide as a glycan donor. In addition, the results show that the PglI-associated acetylation reaction and PglE glycosyltransferase each have relaxed substrate specificities, because they can utilize both Hex and HexNAc as acceptors. These results directly illustrate how biosynthetic components from seemingly diverse pathways can be combined to generate novel glycan structures.

## Discussion

Mutational studies in pathogenic neisseria species have demonstrated the influence of individual *pgl* genes on pilin glycosylation status, but until now there were only suggestive data as to their exact roles and potential interactions. Here, combined reverse genetic and structural approaches have provided the first direct evidence as to the roles of the *N. gonorrhoeae* Pgl components.

**Fig. 10:**
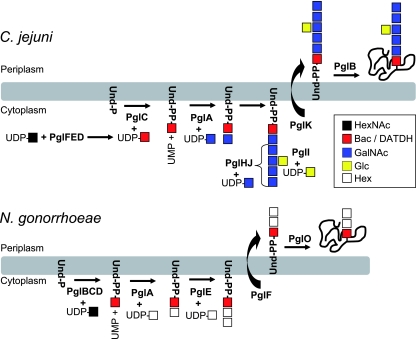
Current model of the pilin glycosylation pathway in *N. gonorrhoeae*. Upper part shows the current model of the *N*-linked glycosylation pathway in *C. jejuni* (Weerapana and Imperiali, 2006). Lower part shows our current model of the *O*-linked glycosylation system in *N. gonorrhoeae*. HexNAc, *N*-acetylated hexose; Bac, bacillosamine; Glc, glucose; Hex, hexose; UMP, uridine monophosphate; Und-PP, undecaprenyl-pyrophosphate.

Based on these results and earlier findings, we propose a neisserial pilin glycosylation pathway which parallels that proposed for the *C. jejuni N*-linked protein glycosylation system (Fig. 10). The most obvious commonality involves components implicated in biosynthesis of the basal DATDH sugar. Despite the unambiguous nature of our findings, the identity of the *N. gonorrhoeae* pilin basal sugar as DATDH remains somewhat contentious, because the presence of at least two other saccharides (GlcNac and 2,4-diacetamido-2,4-dideoxy-β-D-glucopyranoside) at this position has been suggested (Parge *et al*., 1995; Craig *et al*., 2006). Surprisingly, the claims for these alternate sugars were derived from studies of the same strain employed here. Although the basis for these discrepancies has yet to be resolved, our findings here are unequivocal, as both other sugars have molecular masses and fragmentation products distinct from DATDH.

Using purified proteins and defined target substrates, recent studies show that *C. jejuni* PglF, PglE and PglD (acting as a dehydratase, an aminotransferase and an *N*-acetylase respectively) are required for the synthesis of UDP-bacillosamine from UDP-GlcNAc (Olivier *et al*., 2006; Schoenhofen *et al*., 2006). Transfer of bacillosamine from the UDP-sugar to the lipid carrier undecaprenyl phosphate is then accomplished by pgIC acting as a bacillosamine 1-phosphoryl transferase (Glover *et al*., 2006). The roles of *C. jejuni* PglF, PglE, PglD and pgIC at these steps are further corroborated by the alternate incorporation of a WecA-dependent HexNAc as the *N*-linked basal sugar when the genes for each of these components are individually inactivated in *E. coli* carrying the complete *C. jejuni pgl* locus (Linton *et al*., 2005). The complete absence of the glycan here in the *N. gonorrhoeae pgIC* and *pglD* mutants, together with the structural similarities of their products to members of the DegT aminotransferase and dehydratase protein families respectively, is consistent with roles equivalent to those of *C. jejuni* PglE and PglF. The glycosyltransferase and acetyltransferase activities associated with *C. jejuni* pgIC and PglD respectively have been proposed to be carried out in the neisserial systems by a single polypeptide termed PglB (Kahler *et al*., 2001; Power *et al*., 2003), whose characterization by us will be described elsewhere.

A second point of convergence between the *N. gonorrhoeae* and *C. jejuni* pathways involves the components involved in the translocation of the lipid-linked oligosaccharides across the membrane. In *C. jejuni*, PglK carries out this function, as *pglK*-null mutants have been reported in one instance to be defective in protein glycosylation and related phenotypes (Kelly *et al*., 2006), and because it can functionally substitute for the Wzx translocase in O-antigen LPS biosynthesis in *E. coli* (Alaimo *et al*., 2006). The dramatically diminished level of pilin-linked glycan in the *N. gonorrhoeae pglF* mutant, together with its structural similarities of its product to members of the Wzc/RfbX protein family, is consistent with roles equivalent to those of *C. jejuni* PglK. It is of interest to note here that a hypoglycosylation phenotype was also reported for one *C. jejuni pglK*-null mutant (Alaimo *et al*., 2006). Thus, there must be other as-yet-unidentified proteins that function to partially complement the relevant mutations in each system.

Structural characterization of variant glycans in the *pglA* and *pglE*_*on*_ backgrounds clearly demonstrated the specific roles of the two corresponding glycosyltransferases. These results are far reaching, as *pglA* and *pglE* have been reported to be capable of undergoing phase (on–off) variation due to high-frequency changes in the number of repetitive nucleotide sequence stretches in their ORFs (Jennings *et al*., 1998; Banerjee *et al*., 2002; Power *et al*., 2003). As such, it can now be directly inferred that on–off expression of these genes will modulate transitions between the mono-, di- and trisaccharide forms respectively of the glycan. In *C. jejuni*, analogous oligosaccharide extensions require PglA and PglJ that have been shown to act *in vitro* on undecaprenylpyrophosphate-linked glycan substrates (Glover *et al*., 2005; Linton *et al*., 2005). Taken together with *C. jejuni pglA* complementation results here and the fact that *N. gonorrhoeae* PglA, like its *C. jejuni* counterpart, does not contain obvious transmembrane domains or classical secretion motifs, *N. gonorrhoeae O*-linked pilin glycosylation likely involves *en bloc* transfer from an undecaprenylpyrophosphate-linked oligosaccharide. Similar to the current situation with the *C. jejuni N*-linked protein glycosylation, *en bloc* transfer remains to be formally proven in the gonococcal system, as undecaprenylpyrophosphate-linked oligosaccharides have yet to be identified and characterized.

Further evidence for *en bloc* glycan transfer in this system stems from the complete glycosylation defect in *pglO* mutants. Like *P. aeruginosa* PilO, *N. gonorrhoeae* PglO contains the PD416824 domain found in members of the O-antigen ligase/WaaL proteins implicated in transfer of O-antigen oligosaccharides from lipid-linked precursors onto the core elements of LPS. These proteins share minimal sequence similarity, but all are predicted to be integral membrane proteins with comparably distributed membrane-spanning domains. There are limited biochemical data available on WaaL proteins, and as such, the molecular mechanisms underlying their function remain uncharacterized. Topology modelling and membrane-spanning domain organization predict the PD416824 domain to be oriented in the periplasm (Daley *et al*., 2005; Schild *et al*., 2005), the site at which ligation of O-polysaccharide to the lipid A-core occurs (Mulford and Osborn, 1983). Schild *et al*. (2005) identified two conserved motifs in the putative periplasmic domains of *Vibrio cholerae* WaaL proteins as R*X*_3_L and H*X*_10_G located (corresponding to Arg^186^ and His^311^ in loops III and IV of *Vibrio cholerae* WaaL^01^). The R*X*_3_L motif is also recognizable in *N. gonorrhoeae* PglO (data not shown), and the H*X*_10_G motif is encompassed in the HN*X*_2_L*X*_6_G*X*_2_G defined here. As disrupting the integrity of this latter motif in both the *V. cholerae* WaaL protein and *N. gonorrhoeae* PglO abrogates function, we propose that these conserved elements function similarly in oligosaccharyltransferases targeting ligation to both LPS core elements and proteins. Nonetheless, we cannot at this point rule out that the possibility that the PglO sequence alterations made here merely perturb protein stability. Implicit in a lipid-linked oligosaccharide model is the requirement for PglO to function with mono-, di- and trisaccharide precursors. There are clear precedents for such a relaxed specificity, as both WaaL proteins and *P. aeruginosa* PilO have been shown to utilize lipid-associated precursors with varying oligosaccharide chain lengths, including monosaccharide forms (Feldman *et al*., 1999; Horzempa *et al*., 2006).

Another facet of this work connecting pilin modifications to those of LPS is the discovery of glycan *O*-acetylation. Definitive chemical and structural methods showed stoichiometric *O*-acetylation of the proximal hexose, although we did not identify which carbon positions were involved. In fact, CID MS/MS analyses of less prominent oxonium ions from both wild-type and *C. jejuni pglA*-complemented PilE preparations revealed the presence of signals that can best be reconciled as double-acetylated forms of Hex and HexNAc respectively (Fig. S5). Based on its high degree of identity with known LPS acyltransferases (Fig. S6), the association of PglI with this modification likely reflects its activity as a membrane-bound acyltransferase using acetyl-CoA as a donor and pilin glycan as a substrate. It is worth mentioning here that the characteristic sensitivity of *O*-acetyl groups to alkaline conditions likely accounts for the failure for this modification to be detected in ‘bottom-up’ type MS studies (Hegge *et al*., 2004) and earlier structural studies (Parge *et al*., 1995; Craig *et al*., 2006), as tryptic digestions occur under mild alkaline pH and standard purification schemes entail shearing pili off in high-pH buffers. *O*-acetylation can profoundly affect the efficacy of antibody responses towards LPS and CPS (Berry *et al*., 2002; Fox *et al*., 2005) and innate immune responses to peptidoglycan (Boneca *et al*., 2007). Despite extensive immunogenicity and antigenicity studies of gonococcal pili associated with human vaccine trials, the potential influence of pilin glycosylation on such biological activities has yet to be addressed.

We previously demonstrated that the PptA enzyme required for direct, covalent modification of pilin with PE is structurally related to those required for the identical modification of LPS core elements (Hegge *et al*., 2004). Together with the results here for PglI and PglO, these observations provide three distinct examples mechanistically linking *O*-linked polypeptide modifications in *N. gonorrhoeae* to analogous modifications of saccharolipid glycans in LPS. How the pilin-modifying enzymes evolved from their related counterparts, and what selective benefits accrued from these recruitments, are topics for future study.

In conclusion, this work defines the pilin-linked glycan of *N. gonorrhoeae* strain N400 and provides the first conclusive evidence for the roles of individual proteins in glycan biosynthesis, translocation and transfer to pilin. Taken together with prior evidence for potential on–off expression of some *pgl* genes, the results also delineate the glycan repertoire capable of being modulated by a single strain. Given the high degree of *pgl* gene conservation, these findings are undoubtedly applicable to the related *N. meningitidis pgl* systems. The data also provide substantial evidence for functional and structural commonalities linking this pathway to those in the *C. jejuni N*-linked glycosylation and the *P. aeruginosa O*-linked pilin glycosylation systems. In fact, the *N. gonorrhoeae* pathway most closely resembles a hybrid system composed of the DATDH-based, donor oligosaccharide found in the *C. jejuni N*-linked system and an oligosaccharyltransferase of the type seen in the *P. aeruginosa O*-linked pilin system. This work thus vividly illustrates the utility and potential of combinatorial glycoengineering strategies from both evolutionary and applied perspectives.

## Experimental procedures

### Bacterial strains and plasmids

The bacterial strains used in this study are described in Table 1. *E. coli* and gonococcal strains were grown as described (Freitag *et al*., 1995). The *N. gonorrohoeae pglA*, *pgIC*, *pglD* and *pglF* mutants have been previously described (Hegge *et al*., 2004). *E. coli* HB101 and TOP10 (Invitrogen) was used for plasmid propagation and cloning experiments. pUP6 is a derivative of pHSS6 that carries two gonococcal DNA uptake sequences (Wolfgang *et al*., 2000). p2/16/1 is a derivative of pUP6 carrying *iga*, the gene encoding IgA1 protease, and the *ermC* gene and promoter (Wolfgang *et al*., 2000).

**Table 1:** *N. gonorrhoeae* strains used in this study.

Strain	Parent strain	Relevant genotype	S^63^ modification	Reference
N400	VD300	*recA6*(*tetM*)^a^	AcHexDATDH	Tonjum *et al*. (1995)
GGA	N400	*pglA*::*erm*	DATDH	Hegge *et al*. (2004)
KS140	GGA	*pglA*::*erm*, *pglI*::*kan*	DATDH	This work
KS141	N400	*pglA*::*kan*	DATDH	This work
GGC	N400	*pgIC*::*kan*	–	Hegge et al. (2004)
GGD	N400	*pglD*::*kan*	–	Hegge *et al*. (2004)
KS142	N400	*pglE*_*on*_	HexAcHexDATDH	This work
KS143	KS142	*pglE*_*on*_, *pglA*::*kan*	DATDH	This work
GGF	N400	*pglF*::*kan*	(AcHexDATDH)^b^	Hegge *et al*. (2004)
KS144	N400	*pglI*::*kan*	HexDATDH	This work
KS145	N400	*pglO*::*kan*	–	This work
KS146	N400	Δ*pglO*_347−352_	–	This work
KS147	N400	*ΔpglO*_359-−364_	–	This work
KS148	N400	Δ*pglO*_313−318_^c^	–	This work
KS149	KS141	*pglA*::*kan, iga*::*pglA*_*Cj*_^d^	AcGalNAcDATDH	This work
KS150	KS149	*pglA*::*kan, iga*::*pglA*_*Cj*_, *pglI*_*fs*_^e^	GalNAcDATDH	This work
KS151	KS143	*pglA*::*kan, iga*::*pglA*_*Cj*_, *pglE*_*on*_	HexAcGalNAcDATDH	This work

Ac, acetyl-group; Hex, hexose.

a *recA6* is an IPTG-inducible allele of *recA*.

b Residual amounts of AcHexDATDH.

c This *pglO* allele contains two missense mutations in addition to the in-frame deletion: Ala307Val and Gln324Lys.

d *C. jejuni pglA* translational fusion allele cloned into the *igA* locus of *N. gonorrhoeae*.

e Frameshift mutation in the middle of the *pglI* ORF.

### Construction of *pglA-*, *pglI-* and *pglO-*null alleles

A kanamycin-resistance gene cassette was cloned into an AgeI site in plasmid pPgtA5 (Banerjee *et al*., 2002), generating the plasmid ppgtA5-kan. This mutant allele was introduced into strain N400 (wild-type) by transformation and selection for kanamycin-resistance (50 μg ml^−1^), generating strain KS141 (*pglA*::*kan*).

*pglI* was amplified from strain N400 (wild-type) by using the primers pglI5′EcoRI (5′-CG**GAATTC**AGGCGGCTTCTTTTTATTCACC-3′) and pglI3′BamHI (5′-GCG**GGATCC**TAGACGTTGGGGTATTTGGCTGCC-3′) (restriction sites in bold). The polymerase chain reaction (PCR) product was digested with the restriction enzymes EcoRI and BamHI, and was cloned into pUP6 digested with EcoRI and BamHI, generating the plasmid pUP6pglI. This plasmid was cleaved by the restriction enzyme PvuI (situated 965 bp into the 1871 bp long ORF of *pglI*) and blunted. To make the *pglI*_*fs*_ allele, the cleaved and blunted plasmid was simply religated and transformed by a non-selective method described by Gunn and Stein (1996) into *N. gonorrhoeae* strain KS149. Transformants were identified by PCR using the primers pglI5′EcoRI and pglI3′BamHI and digestion of the resulting PCR product with PvuI. To make a kan^r^*pglI*-null mutant, a kanamycin-resistance gene cassette was cloned into the cleaved and blunted PvuI site of pUP6pglI, generating the plasmid pUP6pglI::kan. The disrupted allele carried on pUP6pglI::kan was introduced into the wild-type strain N400 by transformation and selection for kanamycin-resistance (50 μg ml^−1^), generating the strain KS144 (*pglI*::*kan*).

*pglO* was amplified from strain N400 (wild-type) by using the primers pglO5′EcoRI (5′-CG**GAATTC**CATCCCCTTTACCTTCGCACTC-3′) and pglO3′BamHI (5′-GCG**GGATCC**TTGCTTCTTCCGCCCAAGTCTG-3′) (restriction sites in bold). The PCR product was digested with the restriction enzymes EcoRI and BamHI, and was cloned into pUP6 digested with EcoRI and BamHI, generating the plasmid pUP6pglO. A kanamycin-resistance gene cassette was cloned into a blunted MluI site situated 858 bp into the ORF of *pglO* in pUP6pglO, generating the plasmid pUP6pglO::kan. The altered allele in pUP6pglO::kan was introduced into the wild-type strain N400 by transformation and selection forkanamycin-resistance (50 μg ml^−1^), generating the strain KS145 (*pglO*::*kan*).

### Construction of *pglO* in-frame deletion mutants

*pglO* in-frame deletions were created by PCR-based splicing-by-overlap extension (SOE), such that six codons (313–318, 347–352 and 359–364) were replaced by an unique HindIII restriction site. Each pair of overlapping PCR fragments was sewn together by using the flanking primers pglO5′EcoRI (5′-CG**GAATTC**CATCCCCTTTACCTTCGCACTC-3′) and pglO3′BamHI (5′-GCG**GGATCC**TTGCTTCTTCCGCCCAAGTCTG-3′) (restriction sites in bold). The SOE PCR products were digested with EcoRI and BamHI and cloned into pUP6 digested with EcoRI and BamHI.

To generate the 5′-end fragments for the PCR SOE reaction, the flanking primer pglO5′EcoRI was used in combination with each of the three altering primers pglO_Δ313−318__3′ (5′-GGCAAAACTGTTCCA**AAGCTT**GGCGGACTGGAAGGCG-3′), pglO_Δ347−352__3′ (5′-GCAAGGAGTTGGAG**AAGCTT**GGTGAACAAGGTGC-3′), and pglO_Δ359−364__3′ (5′-GCGGCAACCAGAAG**AAGCTT**TTCTGCAAGGAGTTG-3′) respectively (HindIII restriction site in bold). The overlapping 3′-end fragments were generated by using the flanking primer pglO3′BamHI in combination with each of the altering primers pglO_Δ313−318__5′ (5′-CGCCTTCCAGTCCGCC**AAGCTT**TGGAACAGTTTTGCC-3′), pglO_Δ347−352__5′ (5′-GCACCTTGTTCACC**AAGCTT**CTCCAACTCCTTGC-3′), and pglO_Δ359−364__5′ (5′-CAACTCCTTGCAGAA**AAGCTT**CTTCTGGTTGCCGC-3′) respectively (HindIII restriction site in bold).

The altered alleles were introduced into the wild-type strain N400 by transformation with the pUP6 derivatives and screened for the unique HindIII restriction site polymorphism associated with the mutations. Direct DNA sequencing of PCR products derived from gonococcal transformants was performed at GATC Biotech AG (Konstanz, Germany) to verify the deletions and the absence of any other alterations.

### Construction of N400 derivatives carrying the *pglE* phase-on allele

The FA1090 *pglE*_*on*_ allele (corresponding to NG0207) was PCR amplified from FA1090 genomic DNA using the primers av2100 (5′-CAGTGAAAATCCCGAAGACATC-3′) and av2101 (5′-CGCTGGCCGGACATCGTGTTCC-3′). The PCR product was subsequently purified over a Qiagen PCR purification column according to the manufacturer's specifications. The purified PCR product was transformed by a non-selective method described by Gunn and Stein (1996) into the *N. gonorrhoeae* strain N400. Candidate colonies were restreaked and screened by PCR using primers av1111 (5′-CGTGTCCGGATATCGTCAGGATTAGGATTATTTAG-3′) and av1113 (5′-CCGACAATCCTTGGTTATCCTG-3′). Transformants for the ‘corrected’*pglE* allele were identified based on the reduced size of the PCR product (approximately 280 bp versus 350 bp). Direct DNA sequencing of PCR products derived from gonococcal transformants was performed by GATC Biotech AG (Konstanz, Germany) to verify the correct alterations.

### Expression of *C. jejuni pglA* in *N. gonorrhoeae*

A translational fusion between *N. gonorrhoeae pglA* and *C. jejuni pglA* was generated by PCR-based SOE reaction, such that 400 bp of the chromosomal region upstream of the start codon of *N. gonorrhoeae pglA* was fused to the start codon of *C. jejuni pglA*. To generate the 5′-end fragment for the reaction, genomic DNA from *N. gonorrhoeae* strain N400 was used as a template for the flanking primer pglAMS115′_SacI (5′-ACTAGAGCTCGCACGGCAGCCCTGCTTGTCC-3′) (SacI site underlined) in combination with the primer pglA MS11_tf3′(5′-TCCTATTCTCATAAGGCGGACACCTTGAATATAGGATTGG-3′). The overlapping 3′-end fragments were generated from *C. jejuni* DNA by using the flanking primer pglACj3′_SacI (5′- ACTAGAGCTCGCAATTCATCGCTTAATAACTC-3′) (SacI site underlined) in combination with the primer pglACj_tf5′ (5′-GGTGTCCGCCTTATGAGAATAGGATTTTTATCACATGC-3′).

The overlapping PCR fragments were spliced together by using the flanking primers pglAMS115′_SacI and pglACj3′_SacI. The SOE PCR product was digested with SacI and cloned into plasmid p2/16/1 digested with SacI. The *pglA* fusion allele was introduced into the *igA* locus of strain KS141 (*pglA*::*kan*) by transformation with the p2/16/1 derivative and selection for erythromycin-resistance (8 μg ml^−1^), generating the strain KS149 (*pglA*::*kan*, *iga*::*pglA*_*Cj*_).

### SDS-PAGE and immunoblotting

Procedures for SDS-PAGE, and immunoblotting have been described previously (Freitag *et al*., 1995). PilE was detected by immunoblotting of whole-cell lysates, using rabbit polyclonal antibodies and alkaline phosphatase-coupled goat anti-rabbit antibodies (Tago). PilE-specific sera have been described previously (Drake *et al*., 1997).

### Sample preparation for intact protein MS

Type IV pili were isolated as described by Wolfgang *et al*. (1998) and treated with a methanol/chloroform wash/precipitation procedure, as described (Wessel and Flugge, 1984). Briefly, 100 μl of the aqueous PilE solution at 2–3 mg ml^−1^ protein was diluted 1:3 (v/v) with methanol and mixed briefly. Both 100 μl of CHCl_3_ and 200 μl of water were added consecutively, and were followed each time by a mixing step. Phase separation was achieved by centrifugation (4000 *g*, for 8 min), yielding precipitated PilE at the interface. The upper methanol/water phase was removed, and 400 μl of methanol was added. After mixing, the pellet was recovered by centrifugation (13 000 *g*, 8 min). The pellet was dried for 5 min in the inverted tube before dissolving the sample in 50 μl of water/70% formic acid/acetonitrile 3:1:3 (v/v/v). Samples were subjected immediately to MS analyses or frozen at −80°C.

### ESI-MS analysis of intact PilE

All data were acquired on a quadrupole time-of-flight mass spectrometer (Q-Tof micro, Waters Corporation, Milford, MA, USA) equipped with the standard Z-spray ESI source. Sample solutions were infused into the ESI source at a flow rate of 5 μl min^−1^ using a syringe pump (Cole-Parmer Instrument Company, Model SP 100i, Vernon Hills, USA). The source block temperature was maintained at 80°C. Nitrogen was used as both desolvation and nebulizing gas, with flow rates of 228 and 20 l h^−1^ respectively. MS analyses were performed in the ESI-positive mode with the following parameter settings (parameter names as used in the MassLynx NT software, version 4.0): capillary voltage 3000 V; sample cone voltage 25 V; extraction cone voltage 4.3 V; ion energy 2 V; and collision energy 10 eV. Mass spectral resolution was typically 4000. The MS survey was obtained in a mass range from 180 to 1800 *m/z*. Mass calibration in a mass range of 100–2200 *m/z* was performed using the ES tune mix solution from Agilent (Agilent, Palo Alto, CA, USA). The MS spectra were analysed using the MassLynx software (version 4.0). For deconvolution, spectra were processed with the MaxEnt1 program of the MassLynx software. For MS/MS experiments conducted on the Q-Tof, ion selection was undertaken manually. Fragmentation of selected oxonium ions as well as multiple charged PilE ions was induced by CID in the collision cell using argon as collision gas. The CID gas cell pressure was 1.5 × 10^−3^ mbar, and the collision energy in MS/MS experiments was set to 10 eV for intact PilE multiple charged species or 15 eV for glycan oxonium ions. Scan duration in MS mode as well as in the MS/MS mode was set to 1.0 s. Data were acquired and processed by the MassLynx NT software (version 4.0).

### GC-MS *analysis*

Products released from PilE were identified on a GC-MS system (Micromass Prospec Q, Waters Corporation, Milford, USA) with helium as a carrier gas. After a splitless injection of 2 μl (injector 250°C), the temperature of the DBWAX capillary column (30 m × 0.25 mm × 0.25 μm, Agilent Technologies, Palo Alto, CA, USA) was kept at 60°C for 2 min and then increased to 120°C at a rate of 15°C min^−1^. The scan mode at 25–550 *m/z* (electron-impact ionization) was used for searching and for identification of degradation products, whereas extracted SIC were used for semiquantification.

## Supplementary material

The following supplementary material is available for this article:

Fig. S1. Small amounts of oxonium ion for pilin glycan are detected in a *pglF*-null mutant. ESI mass spectra over a range of 180–1800 *m/z*. A. Strain N400 (wt, wild-type), glycan oxonium ion at *m/z* 433.2 is boxed. B. Strain GGC (*pgIC*::*kan*). C. Strain GGD (*pglD*::*kan*). D. GGF (*pglF*::*kan*). The oxonium ion at *m/z* 433.2 is detected in the magnified conventional MS spectrum (boxed in right panel). A complete list of all oxonium ion species with *m/z* values is found in Table S1.

Fig. S2. *N. gonorrhoeae pglE* maps genetically downstream of *pglA*. A. Deconvoluted molecular weight spectrum from intact PilE ESI-MS analyses showing the pilin species found in a *pglE*_*on*_, *pglA* double mutant (strain KS143 *pglE*_*on*_, *pglA*::*kan*). The asterisks mark peaks representing unknown contamination. A complete list of pilin modifications and masses of the corresponding PilE species is found in Table S1. B. ESI mass spectrum over a range of 180–1800 *m/z*. The oxonium ion for DATDH at *m/z* 229.1 (boxed) is detected. A complete list of all oxonium ion species with *m/z* values and corresponding molecular weight values of all PilE species are found in Table S1.

Fig. S3. *N. gonorrhoeae pglI* maps genetically downstream of *pglA* A. Deconvoluted molecular weight spectrum from intact PilE ESI-MS analysis showing the pilin species found in a *pglI*, *pglA* double mutant (strain KS140, *pglA*::*erm*, *pglI*::*kan*). A complete list of pilin modifications and masses of the corresponding PilE species is found in Table S1. B. ESI mass spectrum over a range of 180–1800 *m/z*. The oxonium ion for DATDH (boxed; *m/z* 229.1) is seen. A complete list of oxonium ions and corresponding *m/z* numbers is found in Table S1.

Fig. S4. *C. jejuni pglA* complements a *pglA*-null mutant in *N. gonorrhoeae*. Conventional ESI mass spectra over a range of 180–1800 *m/z* from (A) strain KS149 (*pglA::kan*, *iga::pglA*_*Cj*_); (B) strain KS150 (*pglA::kan*, *iga::pglA*_*Cj*_, *pglIfs*); and (C) strain KS151 (*pglA::kan*, *iga::pglA*_*Cj*_, *pglE*_*on*_). The Gonococcal pilin glycosylation 623 predominant oxonium ion species are boxed in each panel: (A) *m/z* 474.2, (B) *m/z* 432.2 and (C) *m/z* 636.2. A complete list of all oxonium ion species with *m/z* values is found in Table S1.

Fig. S5. Evidence for double acteylation of the proximal Hex and HexNAc residues in wild-type and KS149 PilE disaccharide moieties. A. CID MS/MS spectrum of the oxonium ion at *m/z* 475.2 (boxed; *m/z* 391.2 plus two times 42 Da, doubly acetylated HexDATDH) from the wild-type strain shows the fragmentation into an oxonium ion species at *m/z* 247.2 (boxed) corresponding to doubly acetylated Hex. B. CID MS/MS spectrum of the oxonium ion at *m/z* 516.2 (boxed; *m/z* 432.2 plus two times 42 Da, doubly acetylated HexNAcDATDH) from strain KS149 (*pglA::kan*, *iga::pglA*_*Cj*_) shows the fragmentation into an oxonium ion species at *m/z* 288.2 (boxed) corresponding to doubly acetylated HexNAc.

Fig. S6. PglI shows a high degree of identity to characterized LPS *O*-acetyltransferases. Upper part. Graphical overview of the domain structure of PglI. Colour codes: green, ProDom family PD274832 (residues 8–70); yellow, Acyl_transf_3 domain PF01757 (residues 8–343); red, transmembrane region (residues 350–372); blue, low-complexity region (residues 573–584). Lower part. Alignment of PglI (NG0065) with the LPS *O*-acetylase Lot3 (*N. meningitidis*, NMA2202) and OafA (*H. influenzae*, HAEIN Y392). Colour codes: yellow background indicates identical residues, while blue background indicates similar residues.

Table 1. A complete list of PilE modifications and corresponding masses or *m/z*.

This material is available as part of the online article from: http://www.blackwell-synergy.com/doi/abs/10.1111/j.1365-2958.2007.05806.x

(This link will take you to the article abstract).
