# Supplementary material for: Neisseria gonorrhoeae O-linked pilin glycosylation: functional analyses define both the biosynthetic pathway and glycan structure
Source: Mol Microbiol. 2007 Aug 1;65(3):607–24. doi: 10.1111/j.1365-2958.2007.05806.x (PMC1976384; doi:10.1111/j.1365-2958.2007.05806.x)
Supplement: Fig. S1. — Small amounts of oxonium ion for pilin glycan are detected in a pglF-null mutant. ESI mass spectra over a range of 180–1800 m/z. A. Strain N400 (wt, wild-type), glycan oxonium ion at m/z 433.2 is boxed. B. Strain GGC (pgIC::kan). C. Strain GGD (pglD::kan). D. GGF (pglF::kan). The oxonium ion at m/z 433.2 is detected in the magnified conventional MS spectrum (boxed in right panel). A complete list of all oxonium ion species with m/z values is found in Table S1. [file mmi0065-0607-s1.pdf]

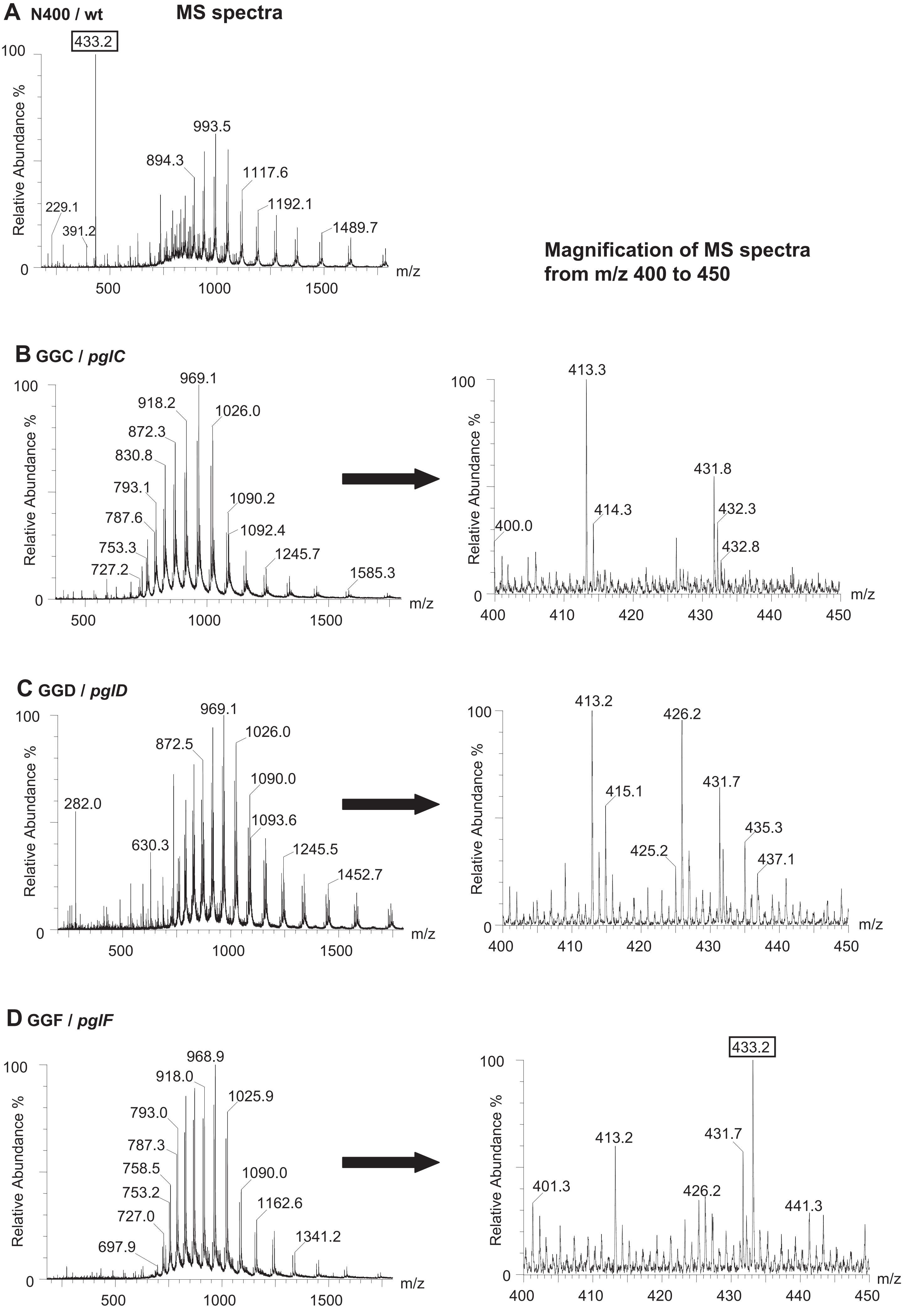
A N400 / wt
MS spectra
433.2
993.5
894.3
1117.6
1192.1
229.1
391.2
1489.7
Relative Abundance %
m/z
Magnification of MS spectra from m/z 400 to 450
B GGC / pglC
969.1
918.2
1026.0
872.3
830.8
793.1
1090.2
787.6
1092.4
753.3
1245.7
727.2
1585.3
413.3
431.8
414.3
432.3
400.0
432.8
C GGD / pglD
969.1
1026.0
872.5
1090.0
282.0
1093.6
630.3
1245.5
1452.7
413.2
426.2
415.1
431.7
435.3
425.2
437.1
D GGF / pglF
968.9
918.0
1025.9
793.0
787.3
758.5
1090.0
753.2
1162.6
727.0
1341.2
697.9
433.2
413.2
431.7
401.3
426.2
441.3

Fig. S1

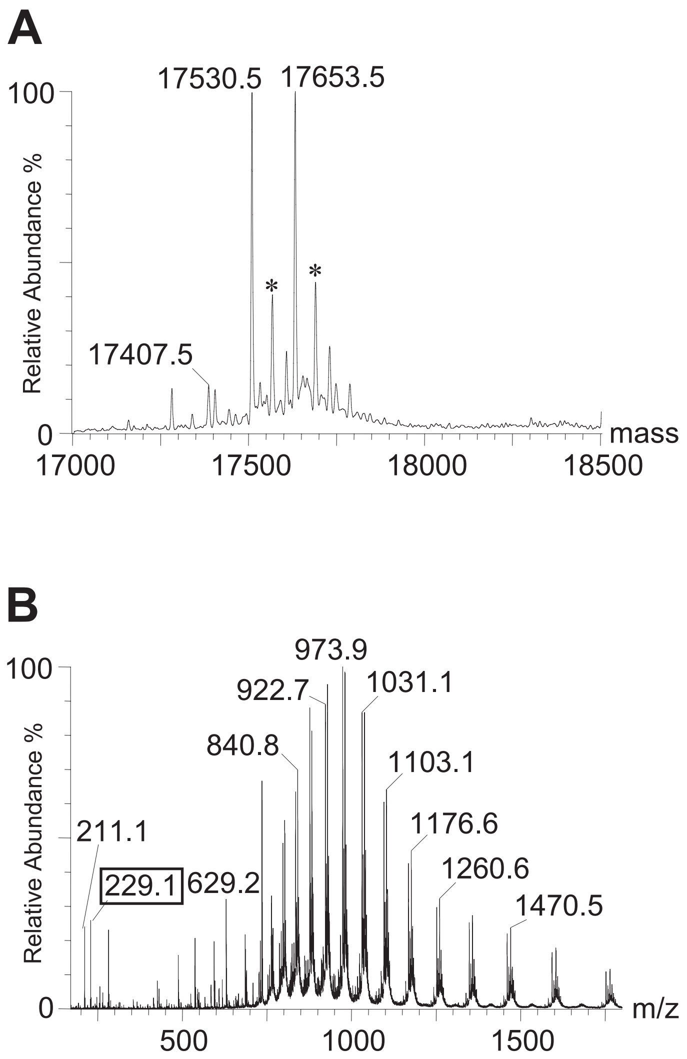
A
17530.5
17653.5
100
Relative Abundance %
*
*
17407.5
0
mass
17000
17500
18000
18500
B
973.9
100
922.7
1031.1
840.8
1103.1
1176.6
211.1
1260.6
229.1
629.2
1470.5
Relative Abundance %
0
m/z
500
1000
1500

Fig. S2

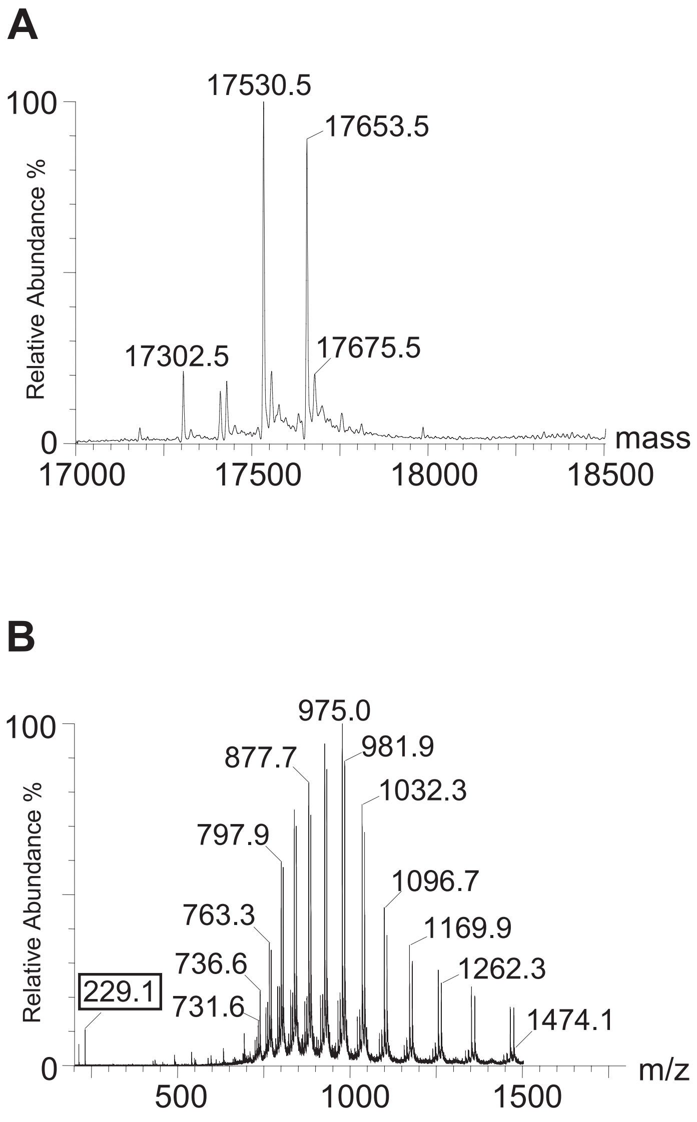
A
100
0
Relative Abundance %
17530.5
17653.5
17302.5
17675.5
mass
17000
17500
18000
18500
B
100
0
Relative Abundance %
975.0
981.9
877.7
1032.3
797.9
1096.7
763.3
1169.9
736.6
1262.3
229.1
731.6
1474.1
m/z
500
1000
1500

Fig. S3

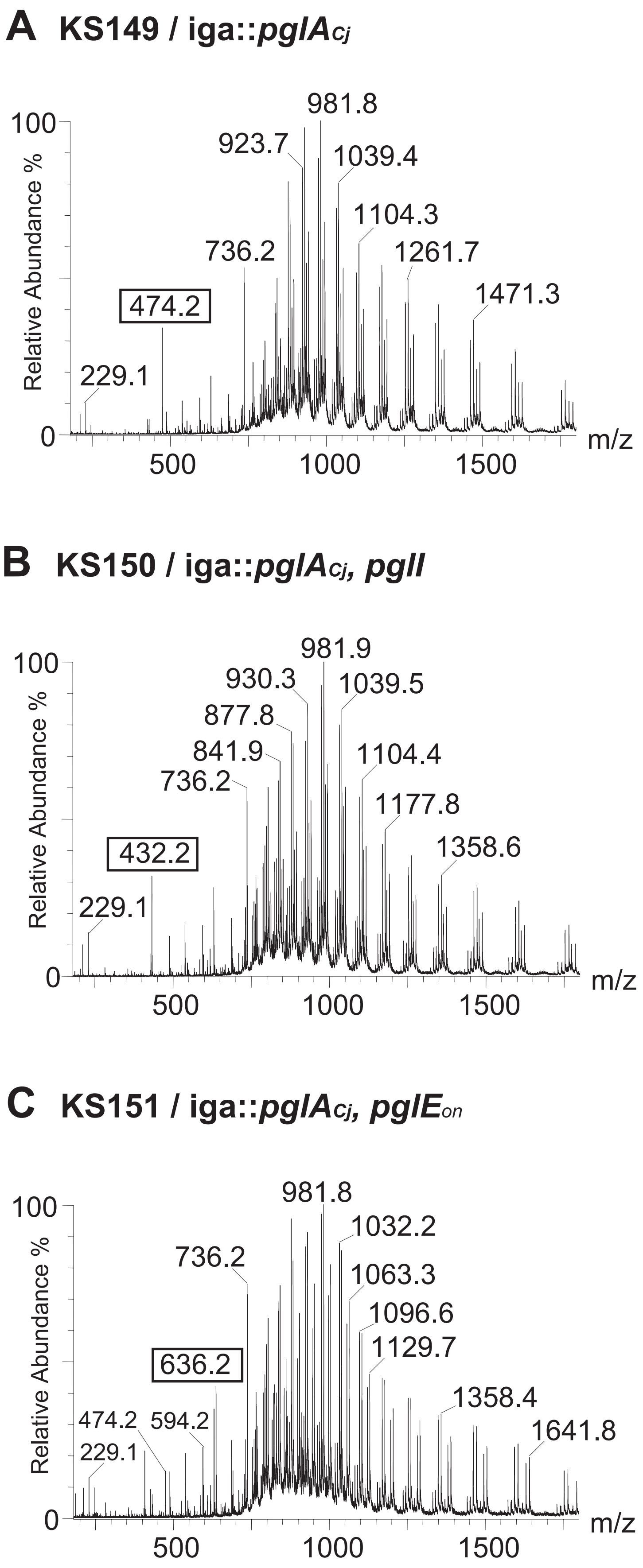
A KS149 / iga::pglACj
100
0
Relative Abundance %
981.8
923.7
1039.4
1104.3
736.2
1261.7
474.2
1471.3
229.1
m/z
500
1000
1500
B KS150 / iga::pglACj, pglI
100
0
Relative Abundance %
981.9
930.3
1039.5
877.8
841.9
1104.4
736.2
1177.8
432.2
1358.6
229.1
m/z
500
1000
1500
C KS151 / iga::pglACj, pglEon
100
0
Relative Abundance %
981.8
1032.2
736.2
1063.3
1096.6
1129.7
636.2
1358.4
474.2
594.2
1641.8
229.1
m/z
500
1000
1500

Fig. S4

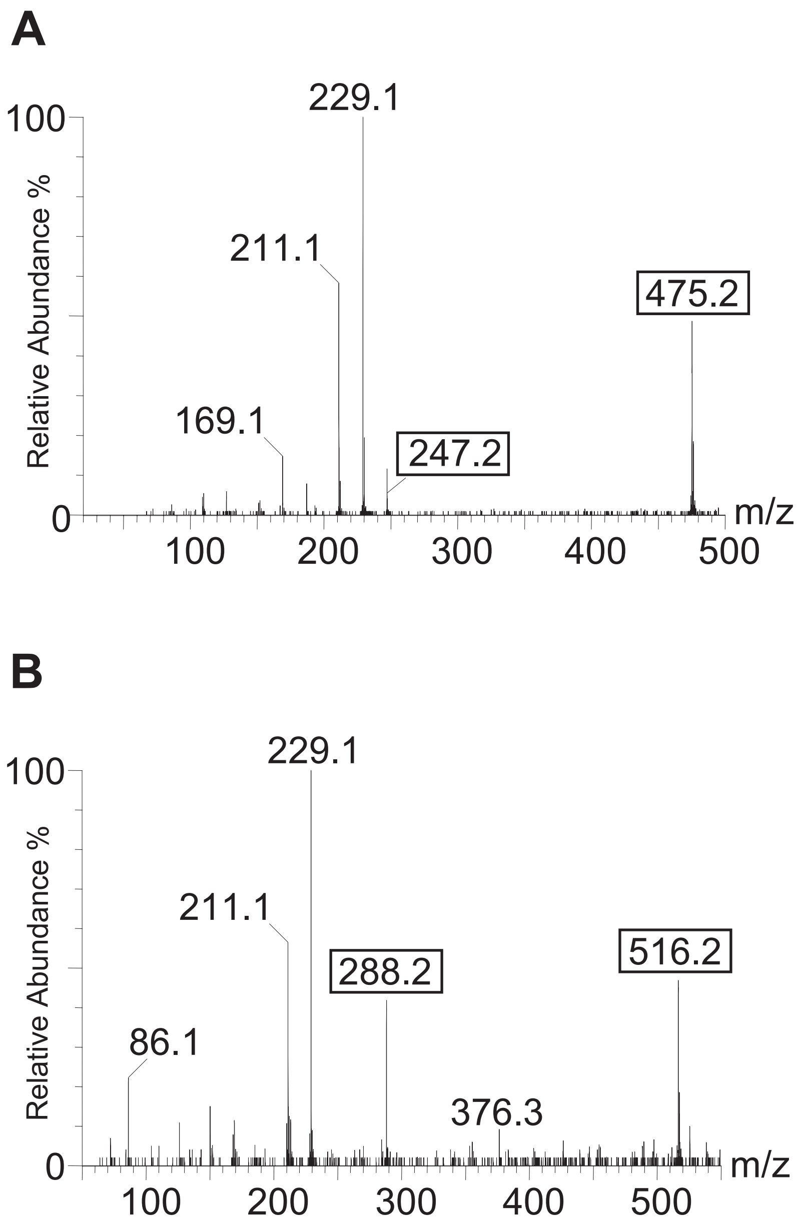
A
229.1
100
Relative Abundance %
211.1
475.2
169.1
247.2
0
m/z
100
200
300
400
500
B
229.1
100
Relative Abundance %
211.1
516.2
288.2
86.1
376.3
0
m/z
100
200
300
400
500

Fig. S5

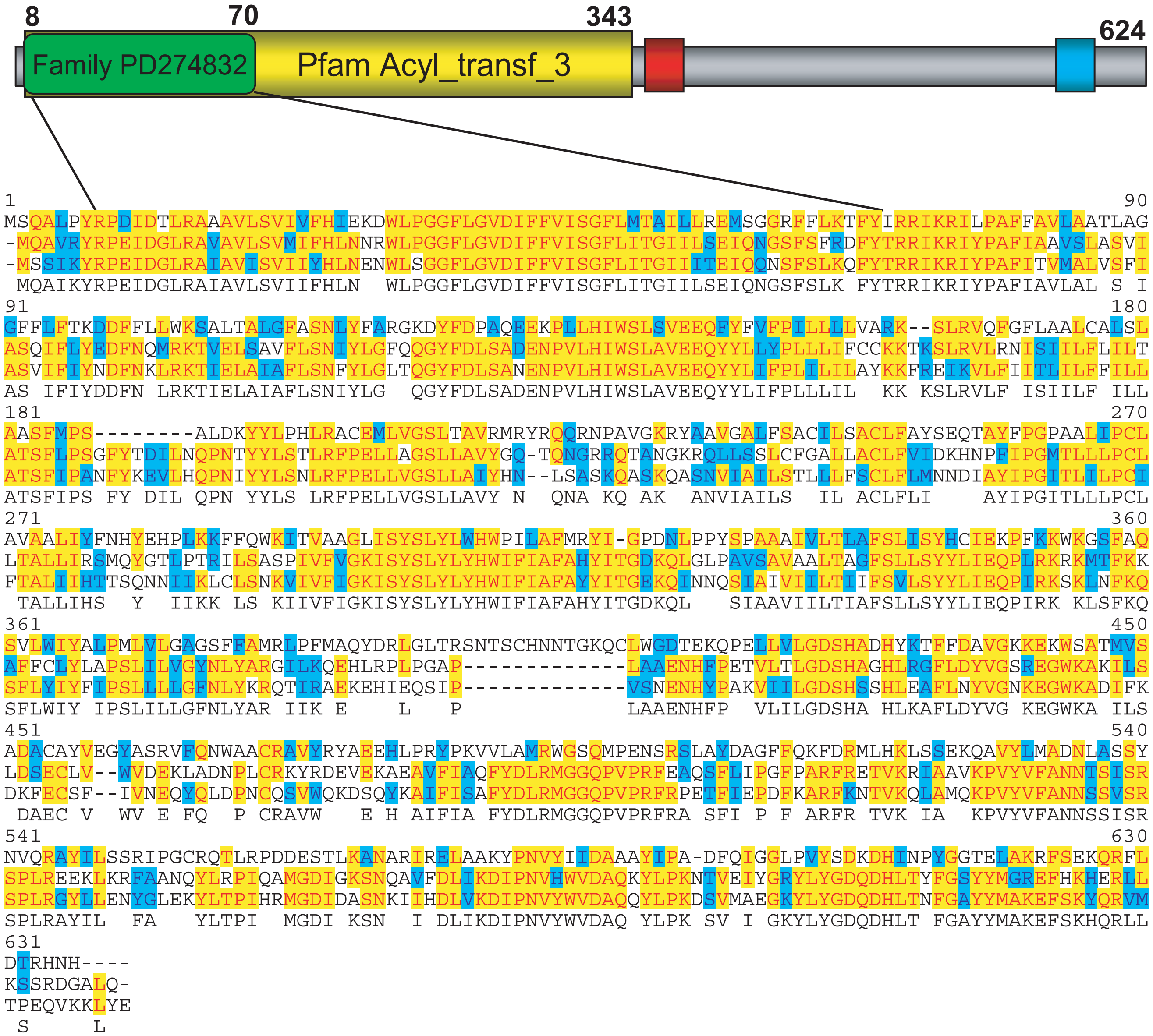


```
                  1                                                                                        90
PglI         (1)  MSQALPYRPDIDTLRAAAVLSVIVFHIEKDWLPGGFLGVDIFFVISGFLMTAILLREMSGGRFFLKTFYIRRIKRILPAFFAVLAATLAG
NMA2202      (1)  -MQAVRYRPEIDGLRAVAVLSVMIFHLNNRWLPGGFLGVDIFFVISGFLITGIILSEIQNGSFSFRDFYTRRIKRIYPAFIAAVSLASVI
HAEIN_Y392   (1)  -MSSIKYRPEIDGLRAIAVISVIIYHLNENWLSGGFLGVDIFFVISGFLITGIIITEIQQNSFSLKQFYTRRIKRIYPAFITVMALVSFI
Consensus    (1)   MQAIKYRPEIDGLRAIAVLSVIIFHLN  WLPGGFLGVDIFFVISGFLITGIILSEIQNGSFSLK FYTRRIKRIYPAFIAVLAL S I
                  91                                                                                      180
PglI        (91)  GFFLFTKDDFFLLWKSALTALGFASNLYFARGKDYFDPAQEEKPLLHIWSLSVEEQFYFVFPILLLLVARK--SLRVQFGFLAALCALSL
NMA2202     (90)  ASQIFLYEDFNQMRKTVELSAVFLSNIYLGFQQGYFDLSADENPVLHIWSLAVEEQYYLLYPLLLIFCCKKTKSLRVLRNISIILFLILT
HAEIN_Y392  (90)  ASVIFIYNDFNKLRKTIELAIAFLSNFYLGLTQGYFDLSANENPVLHIWSLAVEEQYYLIFPLILILAYKKFREIKVLFIITLILFFILL
Consensus   (91)  AS IFIYDDFN LRKTIELAIAFLSNIYLG  QGYFDLSADENPVLHIWSLAVEEQYYLIFPLLLIL  KK KSLRVLF ISIILF ILL
                  181                                                                                     270
PglI       (179)  AASFMPS--------ALDKYYLPHLRACEMLVGSLTAVRMRYRQQRNPAVGKRYAAVGALFSACILSACLFAYSEQTAYFPGPAALIPCL
NMA2202    (180)  ATSFLPSGFYTDILNQPNTYYLSTLRFPELLAGSLLAVYGQ-TQNGRRQTANGKRQLLSSLCFGALLACLFVIDKHNPFIPGMTLLLPCL
HAEIN_Y392 (180)  ATSFIPANFYKEVLHQPNIYYLSNLRFPELLVGSLLAIYHN--LSASKQASKQASNVIAILSTLLLFSCLFLMNNDIAYIPGITLILPCI
Consensus  (181)  ATSFIPS FY DIL QPN YYLS LRFPELLVGSLLAVY N  QNA KQ AK  ANVIAILS  IL ACLFLI     AYIPGITLLLPCL
                  271                                                                                     360
PglI       (261)  AVAALIYFNHYEHPLKKFFQWKITVAAGLISYSLYLWHWPILAFMRYI-GPDNLPPYSPAAAIVLTLAFSLISYHCIEKPFKKWKGSFAQ
NMA2202    (269)  LTALLIRSMQYGTLPTRILSASPIVFVGKISYSLYLYHWIFIAFAHYITGDKQLGLPAVSAVAALTAGFSLLSYYLIEQPLRKRKMTFKK
HAEIN_Y392 (268)  FTALIIHTTSQNNIIKLCLSNKVIVFIGKISYSLYLYHWIFIAFAYYITGEKQINNQSIAIVIILTIIFSVLSYYLIEQPIRKSKLNFKQ
Consensus  (271)   TALLIHS   Y  IIKK LS KIIVFIGKISYSLYLYHWIFIAFAHYITGDKQL   SIAAVIILTIAFSLLSYYLIEQPIRK KLSFKQ
                  361                                                                                     450
PglI       (350)  SVLWIYALPMLVLGAGSFFAMRLPFMAQYDRLGLTRSNTSCHNNTGKQCLWGDTEKQPELLVLGDSHADHYKTFFDAVGKKEKWSATMVS
NMA2202    (359)  AFFCLYLAPSLILVGYNLYARGILKQEHLRPLPGAP-------------LAAENHFPETVLTLGDSHAGHLRGFLDYVGSREGWKAKILS
HAEIN_Y392 (358)  SFLYIYFIPSLLLLGFNLYKRQTIRAEKEHIEQSIP-------------VSNENHYPAKVIILGDSHSSHLEAFLNYVGNKEGWKADIFK
Consensus  (361)  SFLWIY IPSLILLGFNLYAR IIK E    L   P              LAAENHFP  VLILGDSHA HLKAFLDYVG KEGWKA ILS
                  451                                                                                     540
PglI       (440)  ADACAYVEGYASRVFQNWAACRAVYRYAEEHLPRYPKVVLAMRWGSQMPENSRSLAYDAGFFQKFDRMLHKLSSEKQAVYLMADNLASSY
NMA2202    (436)  LDSECLV--WVDEKLADNPLCRKYRDEVEKAEAVFIAQFYDLRMGGQPVPRFEAQSFLIPGFPARFRETVKRIAAVKPVYVFANNTSISR
HAEIN_Y392 (435)  DKFECSF--IVNEQYQLDPNCQSVWQKDSQYKAIFISAFYDLRMGGQPVPRFRPETFIEPDFKARFKNTVKQLAMQKPVYVFANNSSVSR
Consensus  (451)   DAEC V  WV E FQ   P CRAVW    E H AIFIA FYDLRMGGQPVPRFRA SFI P F ARFR TVK IA  KPVYVFANNSSISR
                  541                                                                                     630
PglI       (530)  NVQRAYILSSRIPGCRQTLRPDDESTLKANARIRELAAKYPNVYIIDAAAYIPA-DFQIGGLPVYSDKDHINPYGGTELAKRFSEKQRFL
NMA2202    (524)  SPLREEKLKRFAANQYLRPIQAMGDIGKSNQAVFDLIKDIPNVHWVDAQKYLPKNTVEIYGRYLYGDQDHLTYFGSYYMGREFHKHERLL
HAEIN_Y392 (523)  SPLRGYLLENYGLEKYLTPIHRMGDIDASNKIIHDLVKDIPNVYWVDAQQYLPKDSVMAEGKYLYGDQDHLTNFGAYYMAKEFSKYQRVM
Consensus  (541)  SPLRAYIL   FA    YLTPI  MGDI KSN    I DLIKDIPNVYWVDAQ YLPK SV  I GKYLYGDQDHLT FGAYYMAKEFSKHQRLL
                  631
PglI       (619)  DTRHNH----
NMA2202    (614)  KSSRDGALQ-
HAEIN_Y392 (613)  TPEQVKKLYE
Consensus  (631)   S     L
```

Fig. S6

Supplementary Table 1

A complete list of PilE modifications and corresponding masses / *m/z*.

Modifications present[a]	MW of PilE[b] (Da)
**none**	17179[c]
**1PE**	17302
**2PE**	17425
**DATDH**	17407
**HexDATDH**	17569
**GalNAcDATDH**	17610
**AcHexDATDH**	17611
**AcGalNAcDATDH**	17652
**Hex_2DATDH**	17732
**HexGalNAcDATDH**	17773
**HexAcHexDATDH**	17774
**HexAcGalNAcDATDH**	17815
**1PE, DATDH**	17530
**1PE, HexDATDH**	17692
**1PE, GalNAcDATDH**	17733
**1PE, AcHexDATDH**	17734
**1PE, AcGalNAcDATDH**	17775
**1PE, Hex_2DATDH**	17855
**1PE, HexGalNAcDATDH**	17896
**1PE, HexAcHexDATDH**	17897
**1PE, HexAcGalNAcDATDH**	17938
**2PE, DATDH**	17653
**2PE, HexDATDH**	17815
**2PE, GalNAcDATDH**	17856
**2PE, AcHexDATDH**	17857
**2PE, AcGalNAcDATDH**	17898
**2PE, Hex_2DATDH**	17978
**2PE, HexGalNAcDATDH**	18019
**2PE, HexAcHexDATDH**	18020
**2PE, HexAcGalNAcDATDH**	18061
	***m/z* of Oxonium ion**
**DATDH**	229.1
**Ac_2GalNAc**	288.2
**HexDATDH**	391.1
**GalNAcDATDH**	432.2
**AcHexDATDH**	433.2

Supplementary Table 1 continues

**AcGalNAcDATDH**	474.2
**Ac_2GalNAcDATDH**	516.2
**Hex_2DATDH**	553.2
**HexGalNAcDATDH**	594.2
**HexAcHexDATDH**	595.2
**HexAcGalNAcDATDH**	636.2

[a] PE, phosphoethanolamine; PC, phosphocholine; DATDH, 2,4-diacetamido-2,4,6-trideoxyhexose; Hex, hexose; GalNAc, N-acetyl glucoseamine; Ac, acetyl-group.

[b] Detected or expected MW in ESI MS.

[c] Calculated theoretical MW 17178.5 (including one intramolecular disulfide bridge).

Fig. S1. Small amounts of oxonium ion for pilin glycan is detected in a *pglF* null mutant. ESI mass spectra over a range of 180-1800 *m/z*. A) Strain N400 (wt, wild-type), glycan oxonium ion at *m/z* 433.2 is boxed; B) strain GGC (*pglC::kan*); C) strain GGD (*pglD::kan*); D) GGF (*pglF::kan*). The oxonium ion at *m/z* 433.2 is detected in the magnified conventional MS spectrum (boxed in *right panel*). A complete list of all oxonium ion species with *m/z* values are found in supplemental Table 1.

Fig. S2. *N. gonorrhoeae pglE* maps downstream of *pglA*. A) Deconvoluted molecular weight spectrum from intact PilE ESI mass spectrometric analyses showing the pilin species found in a $pglE_{on}$, *pglA* double mutant (strain KS143 $pglE_{on}$, *pglA::kan*). The asterisks mark peaks representing unknown contamination. A complete list of pilin modifications and masses of the corresponding PilE species is found in supplemental Table 1. B) ESI mass spectrum over a range of 180-1800 *m/z*. The oxonium ion for DATDH at *m/z* 229.1 (boxed) is detected. A complete list of all oxonium ion species with *m/z* values and corresponding molecular weight values of all PilE species are found in supplemental Table 1.

Fig. S3. *N. gonorrhoeae pglI* maps downstream of *pglA*. A) Deconvoluted molecular weight spectrum from intact PilE ESI mass spectrometric analysis showing the pilin species found in a *pglI*, *pglA* double mutant (strain KS140, *pglA::erm, pglI::kan*). A complete list of pilin modifications and masses of the corresponding PilE species is found in supplemental Table 1. B) ESI mass spectrum over a range of 180-1800 m/z. The

oxonium ion for DATDH (boxed; *m/z* 229.1) is seen. A complete list of oxonium ions and corresponding *m/z* numbers is found in supplemental Table 1.

Fig. S4. *C. jejuni pglA* complements a *pglA* null mutant in *N. gonorrhoeae.* Conventional ESI mass spectra over a range of 180-1800 *m/z* from A) strain KS149 (*pglA::kan*, iga::$pglA_{Cj}$); B) strain KS150 (*pglA::kan*, iga::$pglA_{Cj}$, $pglI_{fs}$) and C) strain KS151 (*pglA::kan*, iga::$pglA_{Cj}$, $pglE_{on}$). The predominant oxonium ion species are boxed in each panel; A) *m/z* 474.2, B) *m/z* 432.2 and C) *m/z* 636.2. A complete list of all oxonium ion species with *m/z* values are found in supplemental Table 1.

Fig. S5. Evidence for double acteylation of the proximal Hex and HexNAc residues in wildtype and KS149 PilE disaccharide moieties. A) CID MS/MS spectrum of the oxonium ion at *m/z* 475.2 (boxed; *m/z* 391.2 plus two times 42 Da, doubly acetylated HexDATDH) from the wildtype strain shows the fragmentation into an oxonium ion species at *m/z* 247.2 (boxed) corresponding to doubly acetylated Hex. B) CID MS/MS spectrum of the oxonium ion at *m/z* 516.2 (boxed; *m/z* 432.2 plus two times 42 Da, doubly acetylated HexNAcDATDH) from strain KS149 (*pglA::kan*, iga::$pglA_{Cj}$) shows the fragmentation into an oxonium ion species at *m/z* 288.2 (boxed) corresponding to doubly acetylated HexNAc.

Fig. S6. PglI shows a high degree of identity to characterized LPS *O*-acetyltransferases. *Upper part*. Graphical overview of the domain structure of PglI. Color codes: green, ProDom family PD274832 (residues 8-70); yellow, Acyl_transf_3 domain PF01757

(residues 8-343); red, transmembrane region (residues 350-372); blue, low complexity region (residues 573-584). *Lower part.* Alignment of PglI (NG0065) with the LPS *O*-acetylase Lot3 (*N. meningitidis*, NMA2202) and OafA (*H. influenzae*, HAEIN Y392). Color codes: yellow background indicates identical residues while blue background indicates similar residues.
